# Emerging Interventions in Behavioral Addictions: A Narrative Review of Psychedelics and Neuromodulation

**DOI:** 10.3390/brainsci15090980

**Published:** 2025-09-12

**Authors:** Krista Ulisse, Jehad Albitar, Jourdan T. Aromin, James Berry

**Affiliations:** Department of Behavioral Medicine and Psychiatry, Rockefeller Neuroscience Institute, West Virginia University, Morgantown, WV 26506, USA; krista.ulisse@hsc.wvu.edu (K.U.); jehad.albitar@hsc.wvu.edu (J.A.); jaromin@hsc.wvu.edu (J.T.A.)

**Keywords:** psychedelic, behavioral addiction, compulsive sexual behavior disorder, rTMS, tDCS, gambling disorder, DBS, internet use disorder, neuromodulation, gaming disorder

## Abstract

Addiction remains a persistent public health crisis, marked by poor treatment retention and limited pharmacotherapy options. Emerging treatments, such as psychedelic-assisted psychotherapy and neuromodulation, offer promising avenues for circuit-level interventions in behavioral addictions. This narrative review synthesizes the current landscape of psychedelic compounds and neuromodulation techniques with a focus on their mechanisms of action, applications in specific behavioral addictions, and translational potential. By targeting disrupted reward, executive control, and stress regulation networks, these interventions may facilitate meaningful recovery and long-term remission in otherwise treatment refractory cases. We highlight key findings, current research limitations, and future directions in integrating these novel therapies into the treatment of gambling disorder; internet gaming disorder/gaming disorder, predominantly online; internet use disorder; and compulsive sexual behavior disorder.

## 1. Introduction

Behavioral addictions are chronic, relapsing psychiatric disorders characterized by compulsive engagement in maladaptive behaviors despite significant negative consequences. These conditions share core psychopathological features with substance use disorders—such as impaired impulse control, cravings, and continued engagement despite harm—yet in the absence of a classic intoxicating agent [1,2]. Underlying these conditions are a persistent dysregulation of neural circuits that govern reward processing, motivational drive, stress response, and executive control [3,4]. At the neurobiological level, addiction is conceptualized as a three-phase cycle with the binge/intoxication phase, the withdrawal/negative affect phase, and the preoccupation/anticipation (craving) phase. During the binge/intoxication phase, acute reward intake triggers phasic dopamine release in the mesolimbic (ventral tegmental area → nucleus acumbens) and nigrostriatal (pars compacta → dorsolateral striatum) pathways, reinforcing habit formation [2,3,5]. The withdrawal/negative affect stage follows chronic use, marked by dopaminergic downregulation, glutamate—Gamma-Aminobutyric Acid (GABA) imbalance, and recruitment of stress systems leading to anhedonia and dysphoria [3,6,7,8]. The final preoccupation/anticipation stage involves cue- and stress-induced reinstatement of seeking behaviors, mediated by the prefrontal cortex (PFC), anterior cingulate cortex (ACC), orbitofrontal cortex (OFC), and insular circuits [3,9,10,11]. Together, these stages underpin vulnerability to relapse and chronicity of addiction [3,12]. 

Despite extensive public health initiatives, traditional pharmacological treatments often yield only modest improvements, and relapse rates remain alarmingly high [13,14]. Consequently, novel interventions that directly target neuroplasticity and circuit-level dysfunction have emerged as promising strategies [3,15]. 

The term “psychedelic,” coined in 1957 to mean “mind-manifesting,” describes substances that modulate consciousness via serotonin 5-HT_2_A receptor agonism [16,17]. Chemically, classic psychedelics (psilocybin, Lysergic acid diethylamide [LSD], mescaline, ayahuasca) fall into tryptamine, ergoline, and phenethylamine classes, while dissociative agents (e.g., ketamine, ibogaine) and deliriants represent related categories [18]. Psychedelics are thought to acutely increase prefrontal glutamatergic transmission, flatten hierarchical predictive coding priors, and temporarily reduce top-down constraints on perception and cognition, thereby enabling the revision of rigid maladaptive beliefs and promoting cognitive flexibility and emotional processing [19].

Neuromodulation acts at neurotransmitter levels by re-equilibrating dopamine, serotonin, glutamate, and GABA dynamics [20,21]. It reshapes large-scale brain networks—default mode, salience, and executive-control—to optimize functional connectivity and engages subcortical nodes to restore frontostriatal regulation [22,23,24,25]. Enhanced synaptic plasticity and circuit remodeling via brain-derived neurotrophic factor (BDNF) pathways underpin long-term therapeutic change while improved global efficiency and reduced pathologic clustering reflect normalized network topology [26,27,28]. 

This narrative review critically appraises the literature on the use of psychedelics and neuromodulation in the treatment of gambling disorder; internet gaming disorder/gaming disorder, predominantly online; internet use disorder; and compulsive sexual behavior disorder.

## 2. Methods

A structured literature search was conducted from May 2025 to August 2025 using PubMed, Cochrane Library, Open Evidence, Doximity GPT, Google Scholar, Scopus, APA Psyc Articles, EbscoHost, OVID, Science Direct, and Web of Science databases. The following key words were utilized: 5-MeO-DMT, behavioral addiction, Brodmann area 10 compulsive sexual behavior, compulsive sexual behavior disorder, craving in PPU, DBS, deep brain stimulation, deep TMS, deep transcranial magnetic stimulation, DMT, ECT, electroconvulsive therapy, esketamine, frontopolar cortex, focused ultrasound, gambling, gambling disorder, gaming disorder predominantly online, hypersexuality, impulse control, internet addiction, internet addiction disorder, internet gaming, internet gaming disorder, internet use disorder, ketamine, LSD, mescaline, MDMA, orbitofrontal cortex, pornography, problematic internet use, problematic porn use, problematic pornography use, psilocybin, psychedelic, psychedelic compounds, racemic ketamine, reduction of sexual arousal, repetitive transcranial magnetic simulation, rTMS, sexual addiction, tDCS, theta burst stimulation, TMS, transcranial direct current stimulation, transcranial magnetic stimulation, transcutaneous auricular vagus nerve stimulation, transcutaneous vagus nerve stimulation, TVNS, vagus nerve stimulation, VNS, zona incerta. Studies were screened by three researchers and compiled into the Mendeley Reference Manager for organization. Inclusion criteria are listed as follows: the study mentions behavior or behavioral addictions of interest (compulsive sexual behavior, compulsive sexual behavior disorder, gambling, gambling disorder, gaming disorder predominantly online, hypersexuality, internet addiction, internet addiction disorder, internet gaming disorder, internet use disorder, problematic internet use, problematic pornography use, and sexual addiction) and discusses or applies neuromodulation or psychedelic interventions for treatment/reduction of cravings. Studies that were not published, did not reference the specified behaviors or behavior addictions, or did not discuss neuromodulation or psychedelic interventions were excluded. Date ranges were not specified in exclusion/inclusion criteria given the paucity of research in this area.

## 3. Gambling Disorder

### 3.1. Overview

Gambling disorder is identified in the *Diagnostic and Statistical Manual of Mental Disorders, Fifth Edition, Text Revision* (*DSM-5-TR*) [29], as a persistent and maladaptive pattern of gambling behavior that leads to significant impairment and distress. Data reveal that gambling disorder in the general U.S. population is about 0.2 to 0.3% with a lifetime prevalence of 0.4 to 1.0%, with higher rates among men than women [29]. Gambling disorder is also recognized in the World Health Organization’s (WHO) International Statistical Classification of Diseases and Related Health Problems-11th Revision (ICD-11) [30] as a pattern of persistent or recurrent gambling behavior characterized by impaired control over gambling, prioritization of gambling over other life interests and daily activities, and continuation or escalation of the behavior despite adverse consequences. The ICD-11) further classifies gambling disorder into online and offline subtypes [30]. The online type refers to gambling conducted over electronic networks (e.g., the internet), while the offline type refers to gambling activities that do not involve electronic networks (e.g., not conducted via the internet). Characterized by symptoms such as increasing tolerance and withdrawal-like irritability upon cessation, persistent preoccupation with betting, and “chasing” losses, the disorder shares neurobiological and phenomenological features with substance use disorders [31]. Despite growing recognition of its public health impact on individuals and society, substantial barriers remain in both early identification and access to evidence-based treatments [32]. 

The current first-line treatment is cognitive behavioral therapy (CBT), which has shown strong evidence in the reduction of the severity and intensity of gambling disorder [33]. Other available psychotherapy modalities include motivational interviewing, self-help interventions, and support groups like Gamblers Anonymous [34,35,36]. In terms of medication management, available “off label” treatment options include opioid receptor antagonists (e.g., naltrexone, nalmefene), selective serotonin reuptake inhibitors, mood stabilizers, and atypical antipsychotics [36,37]. However, no medication currently has Food and Drug Administration (FDA) approval for the treatment of gambling disorder, and their effectiveness varies.

Emerging treatments, including psychedelic-assisted therapies and neuromodulation interventions, are being studied for their potential to alter maladaptive reward and control circuits in gambling disorder. Early research targeting the dorsolateral prefrontal cortex (DLPFC) indicates that noninvasive brain stimulation might improve gambling-related decision-making and lessen symptom severity, though findings are mixed and more controlled trials are needed [38,39]. Alongside, psychedelics are being explored as a potential therapy, with initial theoretical and clinical research suggesting potential benefits due to the shared neurobiological mechanisms with other substance use disorders; however, the evidence is still preliminary, and further studies are essential to verify safety and effectiveness in gambling disorder [40]. 

### 3.2. Psychedelics

A recent review of the use of psychedelics in addiction examined multiple studies, including a comparison of LSD and psilocybin microdosing versus no microdosing [41]. LSD may modulate behaviors through the 5-HT_2_A and 5-HT_2_C pathways [42]. Psilocybin is a partial agonist at the 5-HT_2_A receptor that stimulates receptors in cortical and subcortical brain regions, resulting in neurotransmitter release, neural connectivity, changes in the default mode network (DMN), and increased glutamatergic activity. There was no significant difference in problematic gambling behaviors between individuals who microdosed and those who did not microdose [43]. The same review also discussed research from the 1960s investigating psychedelic interventions. One case report evaluated the use of LSD in a patient with both alcohol use disorder and problematic gambling, finding no reduction in either alcohol consumption or gambling with this treatment [44]. Another case report described a patient with problematic gambling who underwent 12 sessions of combined LSD and methylphenidate treatment. The patient initially ceased gambling by the seventh session but subsequently relapsed. However, at a six-month follow-up, the patient reported no further interest in gambling [45]. Brasher et al. [46] found an association between psychedelic use and fewer incidences of problematic gambling; however, there was no association between psychedelic use and well-being in individuals with problematic gambling.

Ketamine has been investigated for its rapid-acting antidepressant properties in and potential for treating various psychiatric disorders, including substance use disorders. Its mechanism, involving N-methyl-D-aspartate (NMDA) receptor antagonism, is believed to modulate reward pathways and reduce compulsive behaviors [47]. While promising, the evidence for ketamine’s efficacy in gambling disorder is limited to an isolated case report, and more clinical trials are needed to establish its efficacy, treatment protocol, and safety profile [48]. 

### 3.3. Transcranial Magnetic Stimulation (TMS)

A variety of high- and low-frequency repetitive-Transcranial Magnetic Stimulation (rTMS) paradigms, as well as continuous theta-burst stimulation (cTBS), have been explored as candidate interventions for gambling disorder, targeting key nodes of executive and inhibitory control networks such as the DLPFC and the pre-supplementary motor area (pre-SMA). Detailed treatment protocols for the referenced studies are outlined in Table 1. In both a case series and a feasibility trial, multiple sessions of high-frequency rTMS over the left DLPFC produced clinically meaningful reductions in gambling severity scales and self-reported days gambled, with effects maintained at 1- to 2-month follow-ups [49,50]. Additionally, a separate single-case report using the same TMS protocol as in the feasibility study was associated with decreased striatal dopamine transporter availability on Single Photon Emission Computed Tomography (SPECT) imaging and sustained abstinence over six months [51]. In contrast, sham-controlled crossover trials using either single-session high-frequency rTMS (HF-rTMS) or low-frequency inhibitory stimulation over prefrontal targets have generally failed to produce durable changes in gambling behavior or craving measures. Transient reductions in cue-induced craving were observed immediately post-stimulation but did not lead to lasting benefit on obsessive-compulsive–style gambling scales [48,52]. Similarly, single-session cTBS applied bilaterally to the pre-SMA at subthreshold intensities did not modulate gambling severity or craving beyond placebo levels [53,54]. Across all modalities, TMS was well tolerated with no serious adverse events reported. Secondary improvements in mood and anxiety noted in open-label protocols were not replicated in randomized designs. Another study using deep TMS (DTMS) delivered via H-coil systems showed robust reductions in depression and anxiety scales, but these mood benefits did not consistently coincide with the behavioral remission of gambling [55]. 

Overall, significant heterogeneity in session schedules (single vs. multi-week), stimulation intensity (80 to 120% of the motor threshold), frequency (1 to 15 Hz versus 50 Hz bursts at 5 Hz for cTBS), and total pulses (360 to 3008 per session) complicates direct comparison across studies. While multi-session high-frequency rTMS over the left DLPFC emerges as the most promising approach for reducing gambling symptoms, single-session and inhibitory paradigms require further optimization. Future large-scale, sham-controlled trials with harmonized stimulation parameters and longer follow-up are essential to establish the efficacy and treatment protocol of TMS in gambling disorder.

### 3.4. Transcranial Direct Current Stimulation (tDCS)

A review examining six studies, with parameters across the studies being similar, using a single 20 to 30-min session at 1.5 to 2 mA intensity demonstrated that tDCS can enhance decision-making and cognitive impulse control, with a broader gain in cognitive flexibility, all of which are disrupted in gambling disorder [58]. However, one study reported gains only being observed in women [59]. Soyata et al. [60] demonstrated improvement in cognitive flexibility in individuals with gambling disorder treated with tDCS (Table 2). A case report published in 2022 targeting the DLPFC found a change in gambling behavior with improved impulsivity control and enhanced decision-making capacity [61]. Another case report noted a positive response to bilateral DLPFC stimulation, with sustained improvement in gambling behavior and co-morbid psychiatric conditions at 3 and 6 months follow up (Table 2) [62]. Additionally, tDCS has been shown to attenuate cravings, as a study (Table 2) found that active tDCS stimulation results in an increase in prefrontal GABA which correlates with reduced risk taking, impulsivity, and craving on behavioral tasks and scales [63]. 

From a mechanistic standpoint, stimulating the DLPFC may help re-balance neural activity between executive and reward systems, potentially correcting the maladaptive decision-making bias observed in gambling. The reviewed evidence supports the therapeutic potential of tDCS as a neuromodulatory tool to enhance cognitive regulation in individuals with gambling-related behaviors, though outcomes vary, and further research is needed to optimize protocols and individualize treatment.

### 3.5. Deep Brain Stimulation (DBS)

There are, to date, no controlled trials of DBS for primary gambling disorder; all published DBS data derive from Parkinson disease (PD) cohorts in whom pathological gambling is most commonly an iatrogenic complication of dopaminergic therapy. In these patients, high-frequency subthalamic nucleus (STN) stimulation appears to exert noticeable effects on reward and impulse-control circuits, sometimes with paradoxical outcomes.

An early case series presented a mixed picture. Ardouin et al. [64] observed remission of pathological gambling in six patients with PD after HF-STN stimulation, but two of these individuals developed postoperative mania that exacerbated gambling, three experienced transient depressive relapses of gambling behavior, and long-term follow-up revealed heightened apathy, with two reaching clinically significant levels. Bandini et al. [65] reported two individuals with PD whose gambling remitted completely within one to two months of combining HF-STN stimulation with marked dopaminergic tapering. Case reports described the emergence or worsening of gambling behaviors following HF-STN stimulation despite reductions in dopamine agonists, an observation that implicates sensitization of limbic pathways by stimulation [66]. Experimental paradigms using gambling tasks corroborate that HF-STN stimulation can alter risk-taking and impulsivity, though they do not evaluate its therapeutic utility in gambling disorder [67]. The study parameters are shown in Table 3.

## 4. Internet Use Disorder and Internet Gaming Disorder/Gaming Disorder, Predominantly Online

### 4.1. Overview

Internet use disorder (IUD), also referred to as internet addiction disorder and problematic internet usage, is a condition that has continued to garner attention in the context of increased utilization, availability, and reliance (both occupationally and socially) upon the internet. Attention to pathological characteristics associated with internet usage began to materialize in the late 1990s [68,69]. Subsequent studies have demonstrated an association between the presence of IUD and other psychiatric comorbidities including depression, anxiety, attention deficit/hyperactivity disorder, alcohol use disorder, and disordered eating [70,71].

Similar to other non-substance behavioral addictions outside of gambling disorder, IUD is not formally recognized in the *DSM-5-TR* nor recognized in the *ICD-11.* However, gaming disorder, predominantly online (GDPO), a condition considered as a subtype of IUD, was included in *ICD-11* in 2018. The diagnostic criteria for GDPO appropriately highlights the importance of considering cultural norms, peer group norms, and subcultures. Preceding the detailed description provided in *ICD-11*, an analogous diagnosis of internet gaming disorder (IGD) was included in the *DSM-5* in 2013. However, this inclusion was placed under the section “Conditions for Further Study.” As the section title indicates, this preliminary term was not intended for formal clinical diagnostic purposes without further continued research. *DSM-5* inclusion criteria for IGD also included elements such as withdrawal, tolerance, preoccupation, deception, and escapism which are not required for GDPO.

Improving technology in neuroimaging has provided further opportunities to assess psychiatric pathology in terms of structure and function in the brain. A prior systematic review by Sepede et al. [72] reviewed functional magnetic resonance imaging (fMRI) studies that included adult patients with IUD with no psychiatric comorbidities. Task related fMRI studies within the analysis demonstrated significant differences in multiple domains within the brain involved in cognitive control and reward processing, similar to findings in substance use disorders. This also included the DLPFC, a popular and feasible noninvasive neuromodulation target. A study by Li et al. [73] utilized fMRI to measure blood oxygen concentration changes in individuals with IUD during decision-making tasks, also noting an association with reduced activation and functional connectivity related to the DLPFC. Multiple studies were found utilizing neuromodulation to target this neural circuitry in IUD and IGD/GDPO, specifically the noninvasive modalities of rTMS and tDCS.

### 4.2. Psychedelics

There were no studies identified using psychedelic compounds for the treatment of IUD and IGD/GDPO. Resting-state functional connectivity (RSFC) and brain networking have been areas of investigation in psychiatric conditions, behavioral addictions, and psychedelic compound mechanisms [74]. A review published in 2022, assessed 16 different network connectivity studies involving administration of LSD or psilocybin [75]. The findings demonstrated a relatively consistent effect of psychedelic compounds on the DMN, specifically a reduction in within-network connectivity accompanied by an increase in between-network connectivity. A more recent review in 2023 also assessed classical psychedelics (including LSD, psilocybin, and ayahuasca) and their potential to modulate the DMN [76]. Recurring observations across these studies again included a reduction in functional connectivity within the DMN with increased connectivity to other resting-state networks, such as the salience network. DMN modulation may serve as a potential mechanism for psychedelic treatment of IUD and GDPO/IGD. A prior study by Wang et al. [77] assessed resting-state fMRI data from 26 adolescents with internet addiction, ultimately demonstrating reduced functional connectivity in the dorsomedial prefrontal cortex of the anterior DMN. There was also reduced functional connectivity between the anterior DMN and the salience network [77].

### 4.3. TMS

Zhong [78] first documented utilization of HF-rTMS for IUD, targeting the DLPFC in a randomized, single blinded, sham-controlled trial. The treatment course for the active treatment arm of the study was 20 sessions daily over the course of 4 weeks, with parameters including stimulation at 100% of motor threshold at 10 Hz for 2000 pulses total each session. The severity of symptoms and cravings were noted to be lower at the end point of the treatment versus the sham. A case report that was published in 2021 by Cuppone [79] included a patient that received HF-rTMS for IGD/GDPO that also targeted the left DLPFC. Parameters, however, were slightly modified- particularly stimulation at 15 Hz for a total of 2400 pulses per session. This patient was also stimulated multiple times per day (2 per day for the first 5 days of treatment) followed by 2 weekly sessions for the next 8 weeks. At the end of the 9-week treatment course, this patient was noted to experience remission of irritability, anxiety, and recurring thoughts about online gaming. This patient also was noted to have no symptoms of internet gaming addiction at a 1 year follow up. A subsequent trial by Chen et al. [80] also utilized HF-rTMS over the left DLPFC in patients with IUD for 40 days versus sham, but the active treatment group also received CBT in addition to HF-rTMS. At the end of the 8 weeks of treatment, the active treatment group had a significant reduction of average Internet Addiction Diagnostic Scale (IAT) scores, Barrett Impulsiveness Scale (BIS-11) scores, and VAS scores versus sham. Another study by Sun et al. [81] also found improvements in symptomatology utilizing HF-rTMS even when a small number of sessions (10 total) was performed over a shorter period (2 weeks) than in the prior studies. 

A more recent study from Hong et al. [82] introduced the concept of attempting to utilize LF-rTMS. One of the two experimental groups consisted of utilizing traditional HF-rTMS to the left DLPFC over 4 weeks, but the second experimental group utilized LF-rTMS to the bilateral DLPFC for one week, followed by HF-rTMS to the left DLPFC in addition to LF-rTMS to the right DLPFC. The findings were suggestive of reduced symptoms utilizing this new protocol. Of note, both experimental groups also initiated sodium valproate during the study, in addition to receiving CBT. Protocol details are outlined in Table 4.

Further evidence is still needed for future clinical applications. There are limitations to the aforementioned TMS studies, most notably for small sample sizes, limited data on long-term outcomes, variable stimulation parameters, differing treatment durations, po-tential operator error in locating the desired target based on proximity to motor cortex versus imaging, and the limited number of randomized, double-blind, sham-controlled studies. Another observed limitation is one study concurrently initiated sodium valproate while initiating TMS. This may alter cortical excitability and subsequently affect a subject’s motor threshold during their treatment.

As previously discussed, reviews of fMRI studies in IUD and IGD/GDPO have demonstrated dysfunction related to the DLPFC and subsequent downstream functional interconnectivity. The selection of the DLPFC as a target for intervention remains common in TMS studies, likely due to its accessibility as a superficial cortical location with a direct role in executive control. Increasing cortical excitability at this target is thought to enhance top-down inhibitory control and cravings—potentially through effects on subcortical structures involved in mesocorticolimbic circuitry—as well as modulating dopaminergic and glutamatergic activity [83,84]. Future considerations include addressing the above study limitations and investigating additional variables of TMS delivery, such as alternative coil design (deep stimulation), theta burst (intermittent and continuous), and accelerated protocols with multiple stimulations per day. The use of alternative coil design that allows deep stimulation may also provide opportunities for more direct targeting of downstream pathways, providing alternatives targets.

### 4.4. tDCS

Trials utilizing tDCS date back even further. Protocol details are outlined in Table 5. Lee et al. [85] completed a prospective single-arm feasibility study. Parameters, like previously discussed HF-rTMS, included a target of the left DLPFC. At the end of the 12 total sessions, there was an observed significant reduction in addiction severity, time spent on games, and cerebral glucose metabolism asymmetry in the DLPFC measured by fluorodeoxyglucose-positron emission tomography (FDG-PET) that was obtained pre-treatment and post-treatment. In 2021, Jeong et al. [86] were able to complete a randomized sham-controlled trial, continuing to target the left DLPFC. This study also demonstrated a significant decrease in time spent gaming, with FGD-PET showing increased cerebral glucose metabolism in the left putamen, pallidum, and insula in the active group versus sham.

Studies released by Wu et al. [87,88] assessed the use of tDCS in men with IGD/GDPO with single-session anodal stimulation over the right DLPFC with a primary focus on assessing negative emotional processing and craving regulation. Findings were notable for improved regulation regarding both cravings and negative emotions versus sham. Their work also suggested right DLPFC anodal stimulation improved inhibitory control over gaming related distractors while also reducing background cravings. However, there was no effect on cue-induced cravings.

A prior study assessed resting-state encephalography (EEG) in individuals with IUD [91]. There was a noted correlation between IUD and higher absolute power on the gamma band, with EEG findings also having an association with impulsivity associated with IUD. Resting-state EEG was later used as an outcome measurement in a randomized, double-blind, sham-controlled parallel group study in 2021 by Lee et al. [85] specifically looking at resting-state EEG spectral activity (absolute power) and functional connectivity (coherence) before tDCS intervention versus one-month post-intervention. The active arm of the study demonstrated a decrease in absolute gamma power associated with the left parietal region versus sham.

There were additional, more recent studies published related to the utilization of tDCS for IUD and IGD/GDPO. One randomized, double-blind, sham-controlled study by Jeong et al. [86] continued to utilize the left DLPFC as the primary target to assess for improvements in inhibitory control in addition to cravings. This study also assessed for potential changes of resting-state functional connectivity (RSFC) in the bilateral DLPFC and nucleus accumbens via fMRI obtained pre-treatment and post-treatment. Significant findings included improved inhibitory control and increased connectivity between the right DLPFC and the ACC. A randomized controlled trial published in 2024 demonstrated continued evolution of neuromodulation technological advances, introducing high definition-tDCS (HD-tDCS) to the IUD and IGD/GDPO literature [89]. The patient population was limited to adolescents (ages 15 to 18) and included a combination active treatment arm of HD-tDCS plus moderate-intensity multimodal exercise. This combination approach (HD-tDCS plus multimodal exercise) demonstrated significant improvements in executive functioning versus monotherapy (HD-tDCS or multimodal exercise alone).

The most recent publication on tDCS with IUD and IGD/GDPO comes from Kim et al. in 2025 [90]. The left DLPFC remained the primary target. Event-related potentials (ERPs) and late positive potentials (LPPs) were utilized to assess for cue reactivity. Patients with IGD/GDPO demonstrated higher LPP amplitudes with game-related cues at baseline versus healthy controls and demonstrated significant decreases in LPP one month after completion of the tDCS intervention.

All reviewed studies utilizing tDCS targeted the DLPFC, similar to findings for rTMS. The consistent selection of the DLPFC for both noninvasive interventional modalities is expected, given that their suspected mechanisms of action are also similar aside from the initial mechanism of stimulation. Low-intensity direct current applied to the DLPFC results in cortical excitability over the target area with subsequent downstream effects in mesocorticolimbic circuitry. As previously discussed, stimulation of the DLPFC is suspected to improve top-down inhibitory control, reduce impulsivity, and decrease cravings [83,84]. Further evidence is still needed to support the clinical use of tDCS. Reviewed studies utilizing tDCS for IUD and IGD/GDPO have similar limitations as discussed in relation to the aforementioned studies assessing rTMS for IUD and IGD/GDPO. This again includes small sample sizes, varying stimulation parameters, limited data on long term outcomes, potential operator error with electrode placement, and a limited number of randomized, double-blind, sham controlled studies. Despite these limitations, tDCS remains an appealing choice for further investigation considering the ease of use and accessibility in the home setting. Future comparison of tDCS versus HD-tDCS should also be considered, as increased complexity of electrode placement may introduce barriers to the practicality of treatment and potentially reduce patient adoption of the modality.

## 5. Compulsive Sexual Behavior Disorder

### 5.1. Overview

Sexual addiction is a highly stigmatized condition that has become increasingly prevalent with the readily accessible nature of online explicit material. The *DSM-5-TR* presently does not presently recognize non-paraphilic hypersexuality as a disorder or condition for future study. In 2019, the *ICD-11* recognized the diagnosis of compulsive sexual behavior disorder (CSBD) [92]. Problematic pornography use (PPU) is the most reported behavioral manifestation of CSBD, although individuals classified as having PPU are not uniformly diagnosed with CSBD [93]. The prevalence of CSBD ranges from 3 to 18% [92]. PPU presents more commonly in males, with a lifetime prevalence of 3 to 10% compared to 1 to 7% in women [94]. Anxiety, depression, other behavioral addictions, and substance use disorders are common psychiatric comorbidities of CSBD [92]. There are no current FDA approved treatments for CSBD. Psychotherapy and off-label medications have been utilized with some effectiveness. Neuromodulation interventions which target cognitive and reward processing circuits—specifically in the brain regions of the SMA, ACC, and DLPFC—and psychedelic-assisted psychotherapy are novel treatment modalities currently being explored for CSBD [79,94,95,96,97,98,99].

### 5.2. Psychedelics

According to the literature, the two primary motivations for compulsive sexual behavior include regulation of cravings and reduction of negative emotions [46,93,94,96]. Psychotherapeutic modalities, particularly CBT and Acceptance and Commitment Therapy (ACT) have demonstrated effectiveness in the reduction of compulsive sexual behavior [46,92,94,96]. Preliminary evidence suggests psychedelics could augment therapeutic processes in the treatment of CSBD by enhancing cognitive flexibility and supporting the integration of one’s sense of self [46,92,93].

Wizła et al. [93] first explored the use of psychedelics in the treatment of CSBD. Abnormalities in the RSFC of the DMN were noted to be similar in CSBD and substance use disorders. The 5-HT_2_A receptors, which are potentiated by LSD, and sigma-1 receptors, which are antagonized by N,N-dimethyltryptamine (DMT), were noted to play important roles in substance-use disorders. Wizła et al. [93] postulated psychedelics, which decrease the resting state activity of the DMN, could allow for the regulation of activity in other networks.

A case report by Strika-Bruneau et al. [92] detailed the clinical improvement of a white, single, French male with CSBD and cannabis use disorder after a recreational use of LSD. After receiving 17 sessions of ACT, he used 150 micrograms of LSD. He reported an increased understanding of ACT principles and their applications to his therapist at their next visit four weeks later. His scores on the sexual addiction questionnaire PATHOS (Preoccupied, Ashamed, Treatment, Hurt others, Out of control, Sad) declined significantly from 6 to 2. Given the clinical cutoff was 3, he no longer met criteria for CSBD. The authors postulated the psychedelic experience led to greater cognitive flexibility which aligned with the goals of ACT. Notably, the psychedelic experience was not supervised by a therapist or recommended by medical professionals and therefore does not qualify as psychedelic-assisted psychotherapy. The authors acknowledge there was no definitive link between the patient’s use of LSD and his symptom improvement.

Brasher et al. [46] examined the use of psychedelic agents, measures of well-being, and symptoms of behavior addictions in a community sample comprised of 1107 participants (67.5% female, 68.4% Caucasian, 55.6% full-time student, 85.3% living in New Jersey, USA) ranging in age from 18 to 79 years. Participants were recruited by e-mail and by undergraduate students and received a link to a survey in Google Forms. The survey included the Problem Gambling Severity Index (PGSI), Sexual Addiction Scale (SAS), Compulsive Buying Index (CBI), SCOFF questionnaire (Sick, Control, One stone, Fat, Food), Growth subscale of the Quiet Ego Scale (GROW), Santa Clara Brief Compassion Scale (BCS3), Patient Health Questionnaire-4 (PHQ-4), Single Item Life Satisfaction (SILS), Single Item Meaning in Life (SIMIL), Single Item Self-Transcendence (SIST), Edinburgh Handedness Inventory (EHI4), and questions detailing substance use history. Data analysis revealed behavioral addiction symptoms were less prevalent in individuals who self-reported a history of psychedelic use, independent of covariates. An increased frequency of psychedelic experiences was associated with a lower frequency of behavioral addiction symptoms. The study population was predominantly female, which is not characteristic of the demographics of individuals with CSBD. Therefore, the generalizability of these results to the population of individuals with CSBD is limited. Additionally, this study population was selected by convenience and measures were self-report, which carries the potential for biases. Causality cannot be inferred given the study design.

The evidence supporting the use of psychedelics in the treatment of CSBD is minimal. There is only one case report noting an association between a recreational ingestion of LSD and a decline in CSBD symptoms [92]. The remainder of the literature discusses theoretical applications of psychedelics in the treatment of CSBD and an association between a low frequency of behavior addiction symptoms in a female population predominantly from New Jersey, USA, that used psychedelics [46,93]. Research in this area is limited given psychedelic use is criminalized in multiple countries. Future research should incorporate a clearly defined protocol for administering psychedelics in a controlled medical setting under the supervision of appropriately trained professionals. Therapists participating in study interventions should have an adequate background in psychedelic assisted psychotherapy. Randomized-control studies with appropriate blinding should be considered to provide the highest quality of evidence.

### 5.3. TMS

In 2016, Tripathi et al. [99] published the first known case report about the use of rTMS in the treatment of hypersexual disorder which was recognized in the *ICD-10*. The patient was a 29-year-old male who was resistant to treatment with multiple trials of antidepressant medications, antipsychotic medications, and electroconvulsive therapy (ECT) and was unable to tolerate depot medroxyprogesterone acetate due to side effects. r-TMS over the SMA was trialed using the parameters outlined in Table 6. After a combination of 4 weeks of rTMS (22 treatments) and escitalopram 20 mg daily, his scores on the Sexual Desire Inventory (SDI) and Sexual Compulsivity Scale (SCS) decreased by 90%. This case report uniquely targeted the SMA which was not targeted in subsequent studies.

Subsequent studies involving rTMS targeted the DLPFC, with parameters outlined in Table 6. In 2020, Schecklmann et al. [98] conducted a randomized, double-blind, sham-controlled crossover study involving 19 healthy males with no psychiatric or medical history to assess if rTMS over the left or right DLPFC reduced their self-reported level of sexual arousal. They hypothesized targeting the right DLPFC would produce the most effects given its role in sexual inhibition. Participants were exposed to 2 control videos and a pornographic video pre- and post- administration of active treatment targeting the right DLPFC, active treatment targeting the left DLPFC, and placebo treatment to the mPFC separated by one-week intervals. Following exposure to each video, participants were administered the Affect and Arousal Scale (AAS), which is a self-report measure assessing perceived sexual arousal. There was a significant reduction of sexual arousal in participants following rTMS targeting the right DLPFC. No significant effects were found with rTMS to the left DLPFC or the sham treatment to the mPFC. In 2021, Cuppone et al. [79] demonstrated conflicting results in a case-report of a 57-year-old married man with CSBD on propranolol for anxiety. The patient demonstrated cessation of cravings for pornography as measured by the VAS after 26 sessions of rTMS to the left DLPFC. Blum and Grant [95] utilized DTMS targeting the ACC) in a 34-year-old male with CSBD confirmed by the Y-BOCS adapted for CSBD. After 28 sessions combined with daily administration of fluoxetine 40 mg, he demonstrated a 39% decrease in symptoms of CSBD as measured by the Y-BOCS adapted for CSBD.

Future research should identify appropriate targets for treatment given the discrepancies in the literature, particularly involving the targeted laterality of the DLPFC. The only randomized control trial to date published involved healthy males who did not meet criteria for CSBD [98]. Additional randomized control trials will be necessary to determine the efficacy of rTMS in the reduction of CSBD symptoms. Deep TMS is another potential therapeutic option that merits additional research given the positive results noted in the published case report [95].

### 5.4. tDCS

In 2023, Dalooyi et al. [96] published a randomized control study involving 9 males between 18 to 24 years old who had PPU and were not receiving other psychological or medication interventions. Participants were randomized into three groups (Table 7), including ACT alone, tDCS targeting the DLPFC (laterality not specified in the study), and ACT combined with tDCS. The study demonstrated that the combination of tDCS and ACT reduced cravings of pornography more than ACT alone and tDCS alone. ACT alone was more efficacious than tDCS alone in reducing pornography cravings. A significant limitation of this study was the minimal available details in English regarding the study methodology, including the number of treatments, stimulation parameters, and specific laterality of the targeted DLPFC.

Sakreida et al. [97] conducted a randomized, double-blind, sham-controlled crossover study (Table 7) assessing the ability of tDCS targeting the right DLPFC and left DLPFC to reduce sexual arousal in 24 healthy heterosexual males with no preexisting psychiatric conditions. Comparable to their group’s study involving rTMS in a similar study population [98], participants were exposed to three separate interventions separated by a week-long interval. Participants viewed two control videos and one pornographic video pre- and post-intervention of tDCS targeting the right or left DLPFC or sham TDCS with electrode placement on the forehead. The AAS was utilized to assess self-reported sexual arousal pre- and post- interventions. There were no significant differences in sexual arousal between the three study conditions.

A double-blind placebo control trial was published by Yang et al. [94] (Table 7). Forty-five male participants determined to be at-risk for PPU (defined as viewing pornography for greater than 6 months at least three times per week on average over the past month and scoring 76 or greater on the Problematic Pornography Consumption Scale) were recruited. Individuals with comorbidities of alcohol use disorder, nicotine use disorder, gaming disorder, gambling disorder, moderate-to-severe depression, moderate-to-severe anxiety, current or previous use of illicit substances, or other neurological or psychiatric disorders were excluded from the study. Participants received both active tDCS targeting the right DLPFC and sham-tDCS separated by one week. Active tDCS significantly reduced pornography cravings; however, it did not significantly enhance the participants’ ability to regulate their emotional states.

Two randomized control studies involving tDCS have been published with conflicting results. However, one study population consisted of healthy individuals with no known psychiatric diagnoses which could confound results. Studies exclusively included male participants. Additional randomized control studies specifically examining the effectiveness of tDCS targeting both the right and left TDCS should be performed with a population more representative of the demographic make-up of individuals with CSBD.

## 6. Discussion

Behavioral addictions are chronic, relapsing and remitting psychiatric disorders with limited treatment options. Advances in neuroscience have led to a greater understanding of the neurobiological mechanisms underpinning behavioral addictions and the potential applications of neuromodulation and psychedelic treatment modalities. Despite current approaches, treatment outcomes remain suboptimal, with high relapse rates and a significant proportion of patients only achieving partial remission. Neuromodulation techniques targeting specific brain circuitry and reward pathways, such as TMS, tDCS, and DBS have emerged as promising potential adjunctive or alternative treatments, particularly for severe and treatment-resistant cases. Concurrently, psychedelics have gained attention for their potential to alter the release and inhibition of neurotransmitters, and their effects on neuroplasticity and brain connectivity. Additionally, their potential to promote psychological insights could support therapeutic techniques targeting the underlying cognitive and emotional patterns of behavioral addictions.

Mechanistically, neuromodulation exerts effects across multiple levels: it modulates neurotransmitter systems (dopamine, serotonin) to reset circuit function, transiently alters cortical excitability thresholds to promote healthy firing patterns, and enhances neuroplasticity via BDNF signaling in both tDCS and rTMS protocols [20,23,26,100,101]. Additionally, tDCS has been shown to increase hippocampal BDNF dependent neurogenesis, supporting cognitive and emotional integration, and to lower pro-inflammatory cytokines (TNF-α, IL-6), mitigating neuroinflammation [22,102,103]. At the network level, these approaches suppress DMN hyperactivity and normalize salience and executive-control connectivity, restoring global functional balance [22,104,105]. Behaviorally, neuromodulation enhances inhibitory control, reduces risk-taking, shifts decision-making toward delayed rewards, and improves executive functions such as working memory and cognitive flexibility, demonstrating translational promise for addiction-related impairments [100,101,106]. Addiction-associated circuit pathologies—hypofrontality, mesolimbic hyperactivity, and maladaptive synaptic remodeling—are thus directly addressed by these interventions [3,26,100].

TMS uses magnetic pulses to change neuronal excitability, thereby rebalancing dysfunctional networks [106]. rTMS exhibits promise in the treatment of gambling disorder, IUD, IGD/GDPO, and CSBD. Deep TMS, which utilizes a specialized coil for targeting, was associated with a decrease in compulsive sexual behavior in a single case study; however, was found to be of no benefit in the treatment of gambling disorder in 5 individuals [55,95]. cTBS has shown inconsistent efficacy in the reduction of gambling behavior [56]. The existing research on TMS is limited by small samples sizes, confounding variables, limited data on long-term outcomes, targeting differences, and variable stimulation parameters. The DLPFC was a popularly targeted area, given its role in decision-making tasks [21]. The frontopolar cortex (FPC, Brodmann area 10), a region of the brain that is involved in exploration-exploitation decision making, may serve as a potential target of TMS meriting further exploration [107]. Individuals with addiction demonstrate an imbalance within the exploration-exploitation dilemma, showing reduced exploration with concurrent increase in exploitation, as evident through continued reward seeking. This shift contributes to reduced impulse control, impaired decision making, impaired learning, and continued perseveration of choice despite consequence—attributes associated with addiction [108]. The orbitofrontal cortex (OFC) should be explored as a future target for cTBS. Studies have demonstrated hyperactivity of the OFC is associated with compulsive behaviors. cTBS would dampen the activity of this region [109,110,111,112]. Randomized, sham-controlled studies with prolonged follow-up would help support the efficacy of rTMS and deep TMS in the treatment of behavioral disorders.

tDCS applies low-intensity electrical currents to modulate resting membrane potentials, enhancing cortical excitability and adaptive plasticity [101,106]. This treatment modality is not approved by the FDA for any recognized diagnosis and is therefore administered off-label or in the research setting. In the referenced studies, tDCS targeting the DLPFC has facilitated an improvement in executive functioning and inhibitory control in the treatment of gambling disorder, IUD, and IGD/GDPO [60,61,62,63,85,86,87,88,89,90]. Results for the application of tDCS in the treatment of CSBD were contradictory [94,96,97]. Future evidence is needed to support the clinical use of tDCS in the treatment of IUD and IGD/GDPO and delineate its potential applications in CSBD. tDCS targeting the frontopolar cortex should be explored in future research, as it has been shown to modulate exploitation-exploration decision making during reward-learning tasks. Increased exploration was associated with anodal stimulation in comparison to increased exploitation with cathodal stimulation [113]. As with TMS, randomized, sham-controlled studies with larger sample sizes are necessary to further support treatment efficacy.

DBS, which targets subcortical structures such as the nucleus accumbens and delivers continuous electrical stimulation to normalize aberrant reward and habit circuits, has been shown to anecdotally decrease gambling behaviors in a cohort of patients with PD with targeting of the STN. However, worsening or emergence of gambling behaviors have also been noted with stimulation to this region [64,65]. The zona incerta (ZI), which is dorsal to the STN and similar to the substantial nigra pars compacta and the ventral tegmental area, is another potential target for future DBS research [114,115]. The ZI is comprised of GABAergic cells, parvalbumin positive neurons, glutamatergic cells, and dopaminergic neurons [115]. Animal studies found an effect of substances of abuse, including alcohol, cocaine, and morphine, on ZI activity [115,116,117,118]. Additionally, ZI neurons facilitate motivation, favor adaptive behavior, and motivate novelty seeking, processes that are implicated in addiction [115]. Future research should examine potential DBS targets in the treatment of behavioral addictions and expand the existing research to include behavioral addictions beyond gambling.

Recent research investigating the therapeutic use of psychedelics has synthesized findings into a conceptual framework across multiple domains, including biochemical, neural, and psychological, to help better understand the complex effects of psychedelics [119]. At a neurotransmitter level, classical psychedelics act as agonists to serotonin 5-HT_2_A receptors, initiating a neural cascade that augments emotional processing and psychological insight [119,120]. Psychedelics have also been associated with rapid enhancements in neuroplasticity and synaptic plasticity, which may underlie their sustained therapeutic benefits [121]. Moreover, psychedelics are linked to an increase in brain entropy, counteracting rigid cognitions and promoting cognitive flexibility and therapeutic change [18,122]. At the psychological level, psychedelics have exhibited their ability to moderate excessive self-focus, which correlates with enhancement in overall well-being [123]. Altered states of consciousness can be induced by psychedelics and manifest in increased introspection, emotionality, creativity, abstract thinking, and a sense of unity [124]. Alteration of the activity of brain networks has been linked with the use of psychedelics, including the DMN, thereby enabling access to emotional and perceptual experiences, facilitating the integration of new insights with pre-existing memories [125]. 

There is currently minimal evidence supporting the use of psychedelics in the treatment of gambling disorder, IUD, IGD/GDPO, and CSBD. However, research in this area is limited. LSD, which acts as an agonist to serotonin receptors, has been demonstrated to affect the DMN and alter decision making processes [42,122]. The evidence for the use of LSD in gambling disorders is mixed, with two studies finding no significant improvement with treatment and one demonstrating remission in symptoms at 6 months after a period of symptom relapse [43,44,45]. An increase in cognitive flexibility and insight into therapeutic principles was exhibited in a patient with CSBD who incidentally used LSD recreationally, however, this did not demonstrate causation [92]. Psilocybin, a partial agonist at the 5-HT_2_A receptor, did not result in a significant reduction of gambling behaviors in a study performed in 2021 [43]. The potential uses of psilocybin in the treatment of IUD, IGD/GDPO, and CSBD have been limited to theoretical exploration in the literature. Ketamine is a non-classical psychedelic in the dissociative subclass which acts via NMDA receptor antagonism and has the potential to modulate reward pathways and reduce compulsive behaviors [47]. One case report supports the efficacy of ketamine’s use for the reduction of gambling behaviors [48]. The reviewed studies have not explored the utilization of ketamine in the treatment of IUD, IGD/GDPO, or CSBD.

Despite promising mechanistic and preliminary clinical data, broader adaptation of psychedelic-assisted therapy requires rigorous large-scale randomized control trials with standardized administration protocols. Logistical challenges, such as the criminalization of psychedelics in many countries, a lack of physicians and therapists trained in psychedelic-assisted treatment, and limited quality control measures to regulate the compounds, pose potential barriers to future research. Regulatory pathways must evolve to accommodate novel treatment methods. Policy initiatives will be needed to enable safe, equitable access to these cutting-edge treatment modalities. Given the high morbidity, mortality, and chronicity associated with behavioral disorders and the limitations of current treatments, there is a continued need for controlled clinical research into novel treatment interventions, such as neuromodulation and psychedelic-assisted therapies.

## Figures and Tables

**Table 1:** Study parameters for publications referencing the use of Transcranial Magnetic Stimulation (TMS) in the treatment of gambling.

Author and Year	N	Technique	Target	Stimulation Parameters	Treatment Number	Duration	Outcomes
Gavazzi et al. [56] 2025	18	cTBS	Pre-SMA	80% of MT, 3 pulses at 50 Hz repeated every 200 ms, 60 s ITI, 1200 total pulses per session	1	1 day	Reduced stop signal reaction times on the Stop Signal Task compared to the untreated controls, demonstrating improved inhibitory control
Cardullo et al. [49] 2019	7	HF-rTMS	Left DLPFC	100% MT, 15 Hz, 4 s train, 15 s ITI, 2400 pulses total per session	26 sessions administered as 2 sessions a day for the first 5 days and then 2 sessions once weekly over 8 weeks	8 weeks	Consistent improvement in G-SAS scores over the 8-week protocol
Gay et al. [57] 2017	22	HF-rTMS	Left DLPFC	110% MT, 10 Hz, 3.2 s train, 10 s ITI, 3008 pulses total per session	1	1 day	Transient reduction in cue-induced craving measured by the VAS. No significant effects on gambling behavior
Pettorruso et al. [51] 2019	1	HF-rTMS	Left DLPFC	100% MT, 15 Hz, 4 s train, 15 s ITI, 2400 total pulses per session	Sessions twice daily, 5 days per week, then a maintenance phase of 2 sessions per week for 12 weeks for a total of 20 treatments	12 weeks	Sustained cessation of gambling behavior and craving at 6 months follow up. Reduced striatal dopamine transporter availability noted at 2 weeks (DAT-SPECT)
Pettorruso et al. [50] 2020	8	HF-rTMS	Left DLPFC	100% MT, 15 Hz, 4 s train, 15 s ITI, 2400 total pulses per session	Sessions twice daily, 5 days per week, then a maintenance phase of 2 sessions per week for 12 weeks for a total of 20 treatments	12 weeks	G-SAS reduced by 71.2%. Improvement sustained throughout the 3-month follow-up
Sauvaget et al. [52] 2018	30	LF-rTMS	Right DLPFC	120% MT, 1 Hz, 1 s train, 360 total pulses per session	1		No differences in craving for gambling between active and sham treatment arms
Rosenberg et al. [55] 2013	5	DTMS	Left DLPFC	110% of MT, 1 Hz, 600 total pulses per session	1 session daily	15 days	All participants continued gambling
Salerno et al. [53] 2022	6	cTBS	Bilateral pre-SMA	80% MT, 3 pulses at 50 Hz repeated every 200 ms, 60 s ITI, 1200 total pulses per session	10 sessions		Improvement of PG-YBOCS scores
Zack et al. [54] 2016	9	HF-rTMS and cTBS	mPFC for HF-rTMS Right DLPFC for cTBS	80% MT, 10 Hz, 3 s train, 10 s ITI, 450 total pulses per session for HF-rTMS 80% MT, 3 pulses at 50 Hz repeated every 200 ms, 5 min ITI, 900 total pulses per session for cTBS	1 session		rTMS: Reduced desire to gamble as measured by the VAS and improved performance on the Stroop task cTBS: Significant improvement in treatment group’s ARCI score compared to sham

Abbreviations: cTBS = continuous Theta-Burst Stimulation; SMA = Supplementary Motor Area; DLPFC = Dorsolateral Prefrontal Cortex; HF-rTMS = High Frequency-repetitive Transcranial Magnetic Stimulation; LF-rTMS = Low Frequency-repetitive Transcranial Magnetic Simulation; rTMS = repetitive Transcranial Magnetic Stimulation; VAS = Visual Analogue Scale; DAT-SPECT = Dopamine Transporter Single-Photon Emission Computed Tomography; G-SAS = Gambling Symptom Assessment Scale; PG-YBOCS = Pathological Gambling Adaptation of the Yale-Brown Obsessive Compulsive Scale; mPFC = Medial Prefrontal Cortex; ITI = Inter-Train Interval; DTMS = Deep Transcranial Magnetic Stimulation; ARCI = Addiction Research Center Inventory.

**Table 2:** Study parameters for publications referencing the use of Transcranial Direct Current Stimulation (tDCS) in the treatment of gambling.

Author and Year	N	Target	Stimulation Parameters	Treatment Number	Outcomes
Soyata et al. [60] 2019	30	DLPFC	Anode applied to the right DLPFC, cathode applied to the left DLPFC. Current of 2 mA applied for 20 min.	3 total treatments administered every other day	Improved cognitive flexibility and decision-making
Dickler et al. [63] 2018	16	DLPFC	Anode applied to the right DLPFC and cathode applied to the left DLPFC. Current of 1 mA applied for 30 min.	2	Increased prefrontal GABA as measured by Magnetic Resonance Spectroscopy
Martinotti et al. [62] 2019	34	DLPFC	Anode applied to the left DLPFC and cathode applied to the right DLPFC. Subsequently, anode applied to the right DLPFC and cathode applied to the left DLPFC. Current of 1.5 mA applied for 20 min.	Daily for 10 days, then weekly for 2 months, then every 2 weeks for 3 months	Significant reduction in cravings as measured by the VAS
Salatino et al. [61] 2021	1	DLPFC	Anode applied to the right DLPFC and cathode applied to the left DLPFC. Current of 1 mA applied for 20 min.	6	Reduction of gambling scores on the SOGS and CPGI

Abbreviations: DLPFC = Dorsolateral Prefrontal Cortex; tDCS = transcranial Direct Current Stimulation; GABA = Gamma-Aminobutyric Acid; VAS = Visual Analogue Scale; SOGS = South Oaks Gambling Scale; CPGI = Canadian Problem Gambling Index.

**Table 3:** Study parameters for publications referencing the use of Deep Brain Stimulation (DBS) in the treatment of gambling.

Author and Year	N	Target	Outcomes
Ardouin et al. [64] 2006	7	STN	Six patients experienced remission; however, two developed postoperative mania that worsened gambling behaviors. Three others had transient depressive episodes that triggered gambling. Long-term follow-up revealed increased apathy across the group, with two patients developing clinically significant apathy.
Bandini et al. [65] 2007	2	STN	Marked reductions in dopaminergic therapy led to a complete resolution of pathological gambling symptoms within one to two months.

Abbreviations: STN = Subthalamic Nucleus.

**Table 4:** Study parameters for publications referencing the use of Transcranial Magnetic Stimulation (TMS) in the treatment of Internet Use Disorder (IUD) and Internet Gaming Disorder (IGD)/Gaming Disorder, Predominantly Online (GDPO).

Author and Year	N	Technique	Target	Stimulation Parameters	Treatment Number	Duration	Outcomes
Zhong [78] 2020	64	HF-rTMS	Left DLPFC	100% MT, 10 Hz, duration of train not provided, ITI not provided, 2000 pulses total per session	20	4 weeks	Addiction severity and background cravings with a greater decline at the end point in the active treatment arm versus sham
Cuppone et al. [79] 2021	1	HF-rTMS	Left DLPFC	100% MT, 15 Hz, 4 s train, 15 s ITI, 1560 pulses total per session	26 sessions administered as 2 sessions a day for the first 5 days and then 2 sessions once weekly over 8 weeks	9 weeks	Significant reduction of time spent on the internet with an improved ability to interrupt internet usage. Continued improvements at 1-year post-intervention
Chen [80] 2022	50	HF-rTMS	Left DLPFC	80% MT, 10 Hz, duration of train not provided, ITI not provided, 1560 pulses total per session	40 sessions administered once daily 5 days per week	8 weeks	Significant reduction of IAT, BIS-11, and VAS in active arm versus control at end point
Sun [81] 2022	61	HF-rTMS	Left DLPFC	100% MT, 10 Hz, duration of train not provided, ITI not provided, 3000 total pulses per session	10 sessions administered once daily 5 days per week	2 weeks	Significantly lower total CIAS score in active treatment arm versus control at end point
Hong [82] 2023	120	HF-rTMS LF-rTMS	Left DLPFC Bilateral DLPFC Right DLPFC	100% MT, 10 Hz, 2 s train duration, ITI 10 s, total pulses per session not provided for HF-rTMS 100% MT, 1 Hz, total pulses per session not provided for LF-rTMS	Experimental Group A: 20 total (1 session daily, 5 days per week over 4 weeks) sessions of HF-rTMS with left DLPFC target Experimental Group B: 5 (1 session for 5 days) bilateral sessions of LF-rTMS with the right DLPFC targeted. This was followed by 15 sessions (1 session daily, 5 days per week over 3 weeks) of HF-rTMS with the left DLPFC targeted in addition to LF-rTMS with the right DLPFC targeted	4 weeks	Both rTMS groups demonstrated a significantly lower YDQ and CIAS score at the end of treatment, with experimental group B demonstrating the lowest scores

Abbreviations: DLPFC = Dorsolateral Prefrontal Cortex; HF-rTMS = High Frequency-repetitive Transcranial Magnetic Stimulation; IAT = Internet Addiction Test; BIS-11 = Barratt Impulsiveness Scale; VAS = Visual Analogue Scale; CIAS = Chen Internet Addiction Scale; LF-rTMS = Low Frequency-repetitive Transcranial Magnetic Stimulation; YDQ = Young Diagnostic Questionnaire.

**Table 5:** Study parameters for publications referencing the use of Transcranial Direct Current Stimulation (tDCS) in the treatment of Internet use Disorder (IUD) and Internet Gaming Disorder (IGD)/Gaming Disorder, Predominantly Online (GDPO).

Author and Year	N	Target	Stimulation Parameters	Treatment Number	Outcomes
Lee [85] 2018	25	DLPFC	Anode applied to the left DLPFC, cathode applied to the right DLPFC. Current of 2 mA applied for 30 min	12 total with 3 sessions per week for 4 weeks	Weekly hours spent playing games, IAT, and BDI-II significantly decreased at endpoint, whereas BSCS increased. The asymmetry index of regional cerebral glucose metabolism of the DLPFC significantly decreased
Jeong, et al. [86] 2021	26	DLPFC	Anode applied to the left DLPFC and cathode applied to the right DLPFC. Current of 2 mA applied for 30 min	12 total with 3 sessions per week for 4 weeks	Significant decrease in time spent on gaming, BIS, BAS-fun seeking, and BAS-reward responsiveness found in active tDCS treatment arm. Significant increase in rCMRglu within the left putamen, palladium, and insula seen in active tDCS treatment arm
Wu [87,88] 2020, 2021	33	DLPFC	Anode applied to the right DLPFC and cathode applied to the left superior region of the trapezius muscle. Current of 1.5 mA applied for 20 min	1 active session and 1 sham session, 1 week apart randomized	Active tDCS facilitated both down regulation and up regulation versus sham; active tDCS reduced interference from gaming-related distractors and attenuated background cravings but not cue-induced cravings
Lee [85] 2021	26	DLPFC	Anode applied to the left DLPFC and cathode applied to the right DLPFC. Current of 2 mA applied for 20 min	10 sessions total with 2 sessions per day for 5 days	Absolute gamma power in left parietal region decreased in active group versus sham at 1 month follow up
Ma [89] 2024	48	DLPFC	HD-tDCS placement with anode and central electrode applied to the left DLPFC. Remaining 4 electrodes applied to F1, FC3, F5, and AF3. Current of 2 mA applied for 20 min	12 sessions total with 1 session per day, 3 days per week for 4 weeks	Combined intervention of HD-tDCS and multimodal exercise significantly outperformed individual interventions/control regarding executive functioning
Kim [90] 2025	32	DLPFC	Anode applied to the left DLPFC and cathode applied to the right DLPFC. Current of 2 mA applied for 20 min	10 sessions total with 2 sessions per day for 5 days	Higher LPP amplitudes with game-related cues at baseline versus healthy controls; significant decrease in LPP one month after completion of tDCS intervention

Abbreviations: DLPFC = Dorsolateral Prefrontal Cortex; tDCS = Transcranial Direct Current Stimulation; IAT = Internet Addiction Test; BDI-II = Beck Depression Inventory-I; BAS = Behavioral Activation System; rCNRglu = Regional Cerebral Metabolism of Glucose; BIS-11 = Barratt Impulsiveness Scale; HD-tDCS = High Definition-Transcranial Direct Current Stimulation; LPP = Late Positive Potentials.

**Table 6:** Study parameters for publications referencing the use of Transcranial Magnetic Stimulation (TMS) in the treatment of Compulsive Sexual Behavior Disorder (CSBD).

Author and Year	N	Technique	Target	Stimulation Parameters	Treatment Number	Duration	Outcomes
Tripathi et al. [99] 2016	1	LF-rTMS	SMA	80% MT, 1 Hz, 5 s ITI, 1120 pulses total per session	22	4 weeks	90% reduction in SDI and SCS scores over 4 weeks with rTMS and escitalopram 20 mg daily
Blum et al. [95] 2020	1	DTMS	ACC	Not provided	28	6 weeks	39% decrease in compulsive sexual behavior with a decrease in Y-BOCS adapted for compulsive sexual behavior from 23 pre-treatment to 14 post-treatment
Schecklmann et al. [98] 2020	19	rTMS	Left DLPFC and right DLPFC	110% MT, 10 Hz, 5 s train interval, 10 s ITI, 3000 pulses total per session	One active treatment targeting the right DLPFC, 1 active treatment targeting the left DLPFC, and 1 placebo treatment separated by 1-week intervals	8 weeks	Significant reduction of sexual arousal measured by the AAS with rTMS to the right DLPFC. No significant effect with rTMS to the left DLPFC
Cuppone et al. [79] 2021	1	rTMS	Left DLPFC	100% MT, 15 Hz, 4 s train, 15 s ITI, 1560 pulses total per session	26 total (2 sessions daily for 5 consecutive days, followed by 2 sessions weekly for the following 8 weeks)	9 weeks	Decrease in VAS measuring cravings for pornography use from 9 pre-treatment to 0 post-treatment

Abbreviations: rTMS = repetitive Transcranial Magnetic Stimulation; SMA = Supplemental Motor Area; MT = Motor Threshold; SDI = Sexual Desire Inventory; SCS = Sexual Compulsivity Scale; DLPFC = Dorsolateral Prefrontal Cortex; ACC = Anterior Cingulate Cortex; TMS = Transcranial Magnetic Stimulation; Y-BOCS = Yale-Brown Obsessive Compulsive Scale; AAS = Affect and Arousal Scale; VAS = Visual Analogue Scale.

**Table 7:** Study parameters for publications referencing the use of Transcranial Direct Current Stimulation (tDCS) in the treatment of Compulsive Sexual Behavior Disorder (CSBD).

Author and Year	N	Target	Stimulation Parameters	Treatment Number	Outcomes
Dalooyi et al. [96] 2023	9	DLPFC (laterality not provided)	Not listed in English	Not listed in English	tDCS + ACT resulted in a significantly greater reduction in cravings and PPU compared to ACT and tDCS alone. ACT and tDCS alone had significant effects on craving reduction and PPU, with ACT alone having a significantly greater reduction than tDCS alone
Sakreida et al. [97] 2023	24	Right DLPFC and left DLPFC	Anode applied to the right DLPFC, anode applied to the left DLPFC, and sham tDCS with electrode placement on the center of the forehead. Cathode placement in all three groups was on the parieto-occipital cortices. Current of 2 mA applied for 20 min.	One active treatment targeting the right DLPFC, one active treatment targeting the left DLPFC, and one placebo treatment separated by one-week intervals	No significant differences in sexual arousal as measured by the AAS between groups
Yang et al. [94] 2025	45	Right DLPFC	Anode applied to the right DLPFC and cathode applied to the supraorbital area. Current of 2.5 mA applied for 20 min.	1 active and 1 placebo treatment separated by one week	Cravings for pornography significantly lower with real stimulation compared to placebo stimulation. No significant differences in emotional regulation with active vs. placebo stimulation

Abbreviations: DLPFC = Dorsolateral Prefrontal Cortex; tDCS = transcranial Direct Current Stimulation; ACT = Acceptance and Commitment Therapy; PPU = Problematic Pornography Use; AAS = Affect and Arousal Scale.

## Data Availability

No new data were created or analyzed in this study.

## Abbreviations

The following abbreviations are used in this manuscript:

AAS	Affect and Arousal Scale
ACC	Anterior Cingulate Cortex
ACT	Acceptance and Commitment Therapy
ARCI	Addiction Research Center Inventory
BAS	Behavior Activation System
BCS3	Santa Clara Brief Compassion Scale
BDI-II	Beck Depression Inventory
BDNF	Brain-Derived Neurotrophic Factor
BIS-11	Barratt Impulsiveness Scale
CBT	Cognitive Behavioral Therapy
CBI	Compulsive Buying Index
CIAS	Chen Internet Addiction Scale
CPGI	Canadian Problem Gambling Index
CSBD	Compulsive Sexual Behavior Disorder
cTBS	Continuous Theta-Burst Stimulation
DAT-SPECT	Dopamine Transporter Single-Photon Emission Computed Tomography
DBS	Deep Brain Stimulation
DLPFC	Dorsolateral Prefrontal Cortex
DMT	N,N-Dimethyltryptamine
DMN	Default Mode Network
DTMS	Deep Transcranial Magnetic Stimulation
DSM	Diagnostic and Statistical Manual
ECT	Electroconvulsive Therapy
EEG	Electroencephalography
EHI4	Edinburgh Handedness Inventory
ERP	Event Related Potentials
FDA	Food and Drug Administration
FDG-PET	Fluorodeoxyglucose-Positron Emission Tomography
fMRI	Functional Magnetic Resonance Imaging
FPC	Frontopolar Cortex
GABA	Gamma-Aminobutyric Acid
GDPO	Gaming Disorder, Predominantly Online
GROW	Growth subscale of the Quiet Ego Scale
G-SAS	Gambling Symptom Assessment Scale
HD-tDCS	High Definition-Transcranial Direct Current Stimulation
HF-rTMS	High Frequency-Repetitive Transcranial Magnetic Stimulation
IAT	Internet Addiction Test
ICD	International Statistical Classification of Diseases and Related Health Problems
IGD	Internet Gaming Disorder
ITI	Inter-Train Interval
IUD	Internet Use Disorder
LF-rTMS	Low Frequency-Repetitive Transcranial Magnetic Simulation
LPP	Late Positive Potentials
LSD	Lysergic acid diethylamide
mPFC	Medial Prefrontal Cortex
MT	Motor Threshold
NMDA	N-methyl-D-aspartate
OFC	Orbitofrontal cortex
PATHOS	Preoccupied, Ashamed, Treatment, Hurt others, Out of control, Sad
PD	Parkinson Disease
PFC	Prefrontal Cortex
PGSI	Problem Gambling Severity Index
PG-YBOCS	Pathological Gambling adaptation of the Yale-Brown Obsessive Compulsive Scale
PHQ	Patient Health Questionnaire
PPU	Problematic Pornography Use
rCMRglu	Regional Cerebral Metabolism of Glucose
RSFC	Regional resting-state functional connectivity
rTMS	Repetitive Transcranial Magnetic Stimulation
SAS	Sexual Addiction Scale
SCS	Sexual Compulsivity Scale
SCOFF	Sick, Control, One stone, Fat, Food
SDI	Sexual Desire Inventory
SILS	Single Item Life Satisfaction
SIMIL	Single Item Meaning in Life
SIST	Single Item Self-Transcendence
SMA	Supplemental Motor Area
SOGS	South Oaks Gambling Scale
SPECT	Single Photon Emission Computed Tomography
STN	Subthalamic Nucleus
tDCS	transcranial Direct Current Stimulation
TMS	Transcranial Magnetic Stimulation
VAS	Visual Analogue Scale
Y-BOCS	Yale-Brown Obsessive Compulsive Scale
YDG	Young Diagnostic Questionnaire
ZI	Zona Incerta
